# Supplementary material for: Phosphorylation of the smooth muscle master splicing regulator RBPMS regulates its splicing activity
Source: Nucleic Acids Res. 2022 Nov 21;50(20):11895–915. doi: 10.1093/nar/gkac1048 (PMC9723635; doi:10.1093/nar/gkac1048)
Supplement: gkac1048_Supplemental_File [file gkac1048_supplemental_file.pdf]

Figure S1

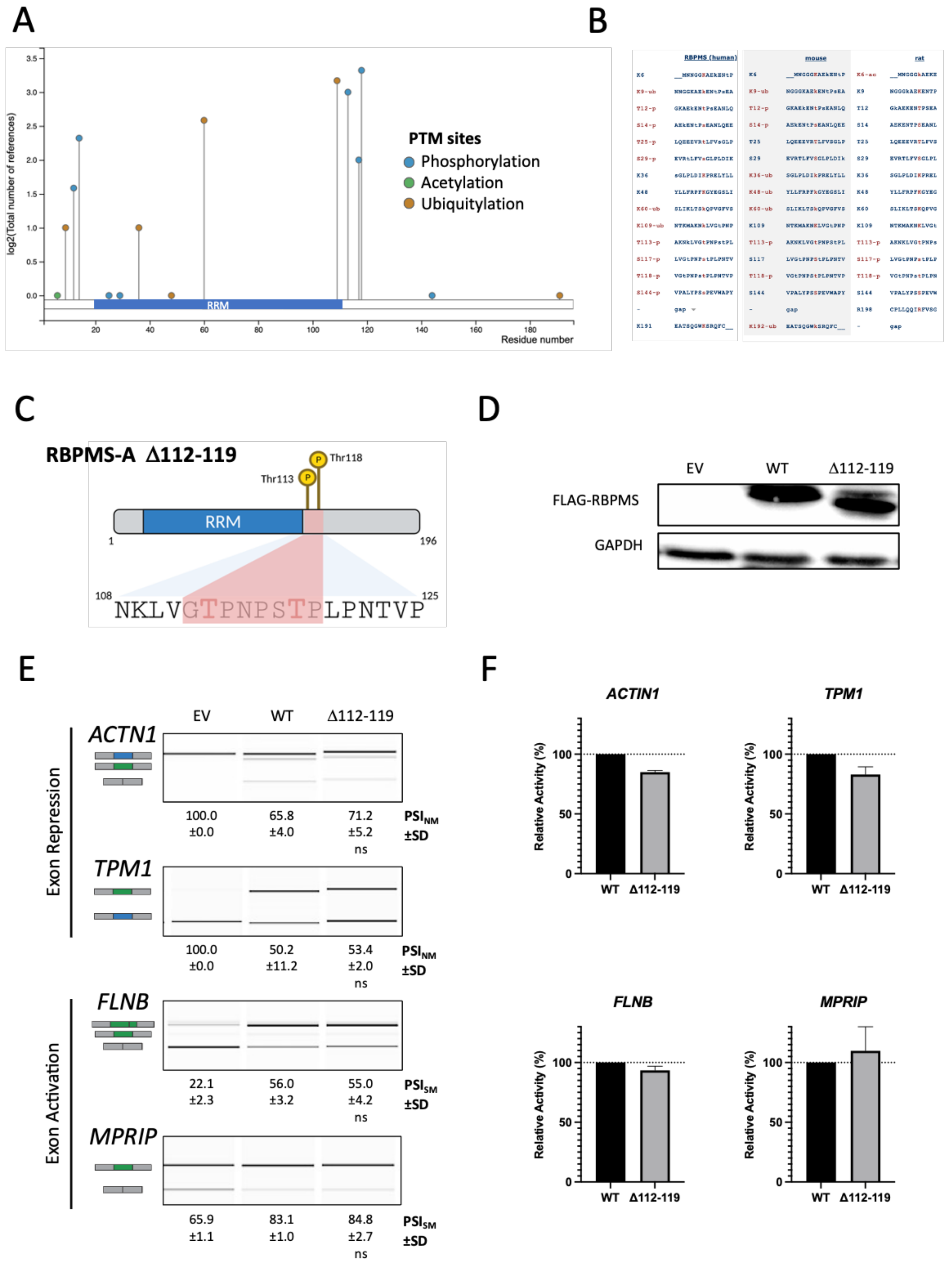
A
B
log2(Total number of references)
PTM sites
Phosphorylation
Acetylation
Ubiquitylation
RRM
Residue number
RBPMS (human)
mouse
rat
C
D
RBPMS-A Δ112-119
Thr118
Thr113
RRM
1
196
108
125
NKLVGTPNPSTPLPNTVP
EV
WT
Δ112-119
FLAG-RBPMS
GAPDH
E
F
EV
WT
Δ112-119
Exon Repression
ACTN1
100.0 ±0.0
65.8 ±4.0
71.2 ±5.2 ns
PSI NM ±SD
TPM1
100.0 ±0.0
50.2 ±11.2
53.4 ±2.0 ns
PSI NM ±SD
Exon Activation
FLNB
22.1 ±2.3
56.0 ±3.2
55.0 ±4.2 ns
PSI SM ±SD
MPRIP
65.9 ±1.1
83.1 ±1.0
84.8 ±2.7 ns
PSI SM ±SD
ACTIN1
TPM1
FLNB
MPRIP
Relative Activity (%)
WT
Δ112-119

Supplementary Figure S1. Post-translational Modifications Identified for RBPMS.

(A) Post-translational modification location along RBPMS and the total number of references (published data sets), with modified RRM to reflect the correct length of the RRM.

(B) Conservation of PTMs across mouse and rat species that have been observed thus far in published data sets. (A&B) Figures modified from (Hornbeck et al, 2015). https://www.phosphosite.org/proteinAction.action?id=2381994

(C) Graphical representation of RBPMS-A with the T113 and T118 highlighted in red. T/A is a double mutant of T113A and T118A, while T/E is a double mutant of T113E and T118E. Created with BioRender.com

(D) Immunoblot of FLAG-RBPMS effectors used for splicing assays in (E). Whole-cell lysates were probed with antibodies to FLAG and GAPDH. GAPDH was used as a loading control.

(E) Splicing analysis of the exon repression function of wild-type (WT) RBPMS-A and effectors in endogenous *ACTN1* and *TPM1*, and its exon activation function in endogenous *FLNB* and *MPRIP* in HEK293T cells. RT-PCR followed by visualization on QIAxcel. Cartoons below the gene names represent the PCR products. The blue exon in the cartoon represents the NM (Non-smooth Muscle) exon, while the green exon represents the SM (Smooth Muscle) exon. PSI (Percent Spliced In) values are the mean ± SD (n = 3). A Student's t-test was used to determine statistical significance between wild-type RBPMS-A and effector. EV=empty vector.

(F) Bar graphs showing the percent relative splicing efficiency of each effector normalized to wild-type RBPMS-A (black bar) after background splicing was taken into consideration. Error bars indicate SD.

Figure S2

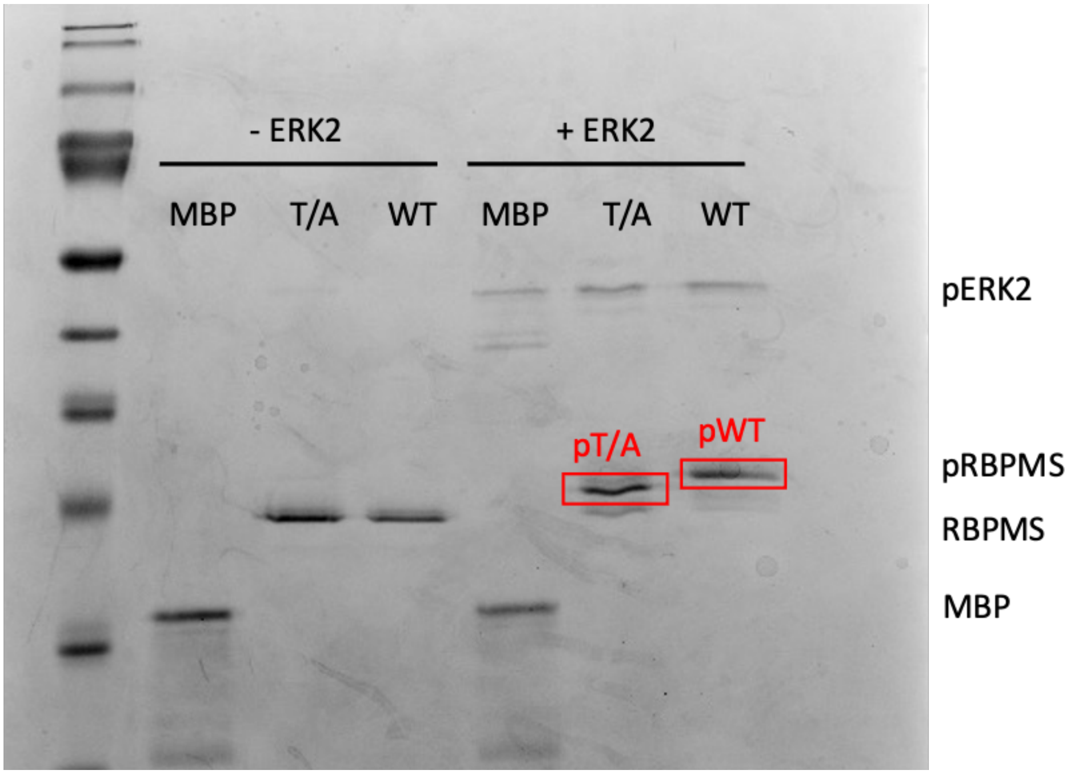

Supplementary Figure S2. ERK2 Phosphorylates RBPMS-A.
Recombinant wild-type (WT) RBPMS-A or RBPMS-A T/A (substrate; 1uM) were incubated with 200 nM highly active recombinant ERK2 in an *in vitro* kinase assay. Reaction was resolved on 20% SDS-PAGE and phosphorylated WT and T/A were excised from the gel (red rectangle) and sent for mass spectrometry. Myelin Basic Protein (MBP) was used as a positive control.

Figure S3

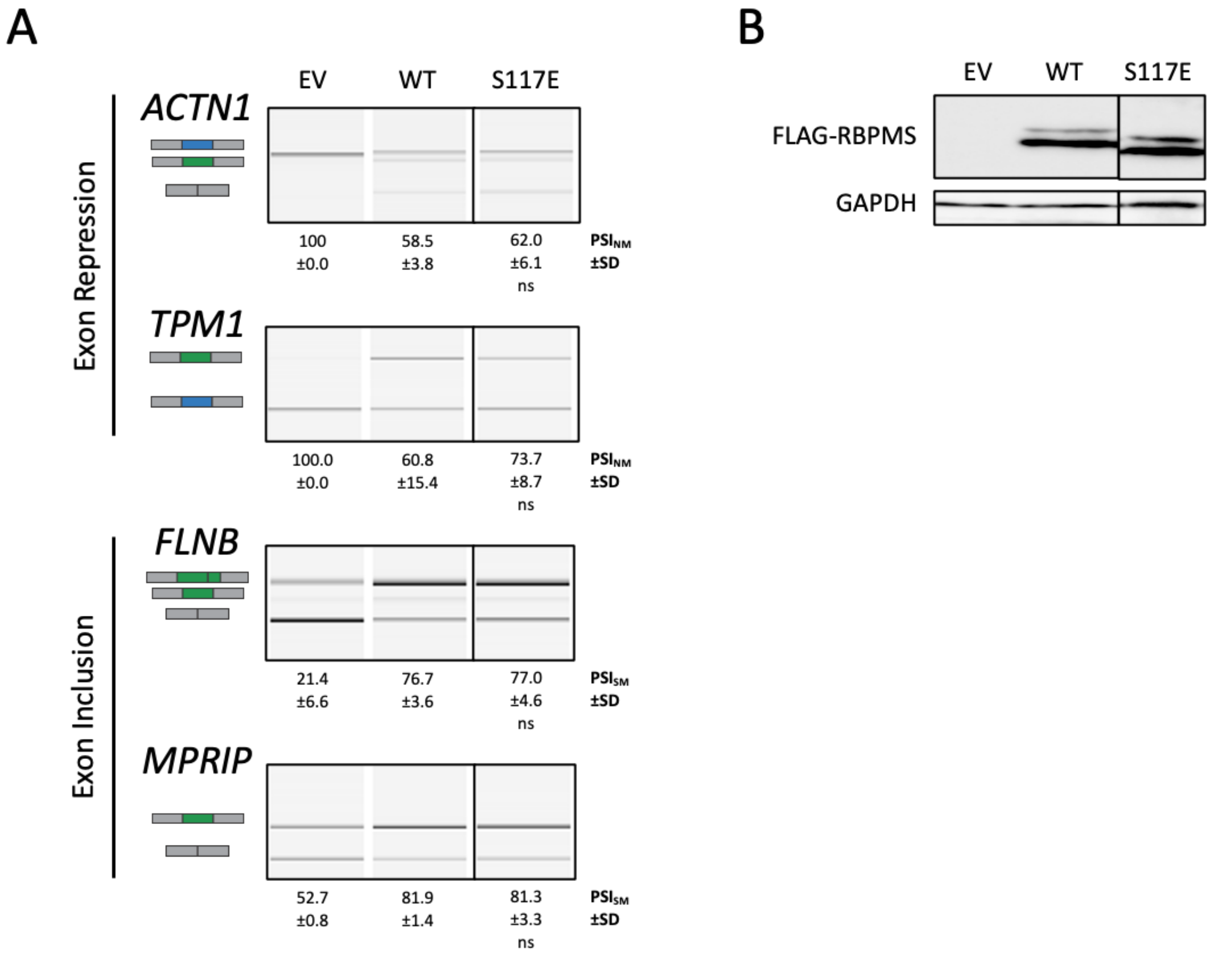

Supplementary Figure S3. S117E Mutation has no Effect on RBPMS-A Splicing Activity.

(A) Splicing analysis of the exon repression function of wild-type (WT) RBPMS-A and effectors in endogenous *ACTN1* and *TPM1*, and its exon activation function in endogenous *FLNB* and *MPRIP* in HEK293T cells. RT-PCR followed by visualization on QIAxcel. Cartoons below the gene names represent the PCR products. The blue exon in the cartoon represents the NM (Non-smooth Muscle) exon, while the green exon represents the SM (Smooth Muscle) exon. PSI (Percent Spliced In) values are the mean ± SD (n = 3). A Student's t-test was used to determine statistical significance between wild-type RBPMS-A and effector. EV=empty vector

(B) Immunoblot of FLAG-RBPMS effectors used for splicing assays in (B). Whole-cell lysates were probed with antibodies to FLAG and GAPDH. GAPDH was used as a loading control.

Figure S4

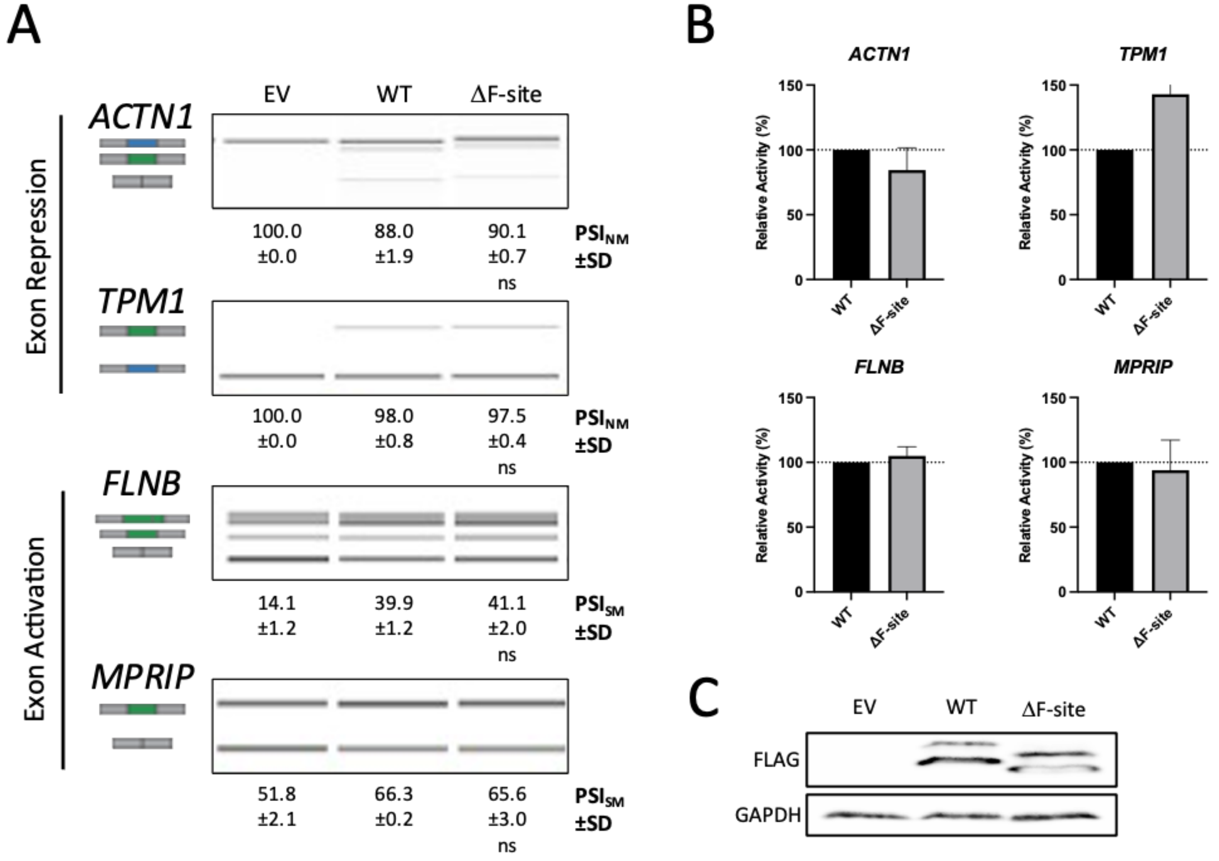

Supplementary Figure S4. Deletion of the ERK2 Docking Site (F-site) has no Effect on Splicing Activity.

(A) Splicing analysis of the exon repression function of wild-type (WT) RBPMS-A and effectors in endogenous *ACTN1* and *TPM1*, and its exon activation function in endogenous *FLNB* and *MPRIP* in HEK293T cells. RT-PCR followed by visualization on QIAxcel. Cartoons below the gene names represent the PCR products. The blue exon in the cartoon represents the NM (Non-smooth Muscle) exon, while the green exon represents the SM (Smooth Muscle) exon. PSI (Percent Spliced In) values are the mean ± SD (n = 3). A Student's t-test was used to determine statistical significance between wild-type RBPMS-A and effector. EV=empty vector.

(B) Bar graphs showing the percent relative splicing efficiency of each effector normalized to wild-type RBPMS-A (black bar) after background splicing was taken into consideration. Error bars indicate SD.

(C) Immunoblot of FLAG-RBPMS effectors used for splicing assays in (C). Whole-cell lysates were probed with antibodies to FLAG and GAPDH. GAPDH was used as a loading control.

Figure S5

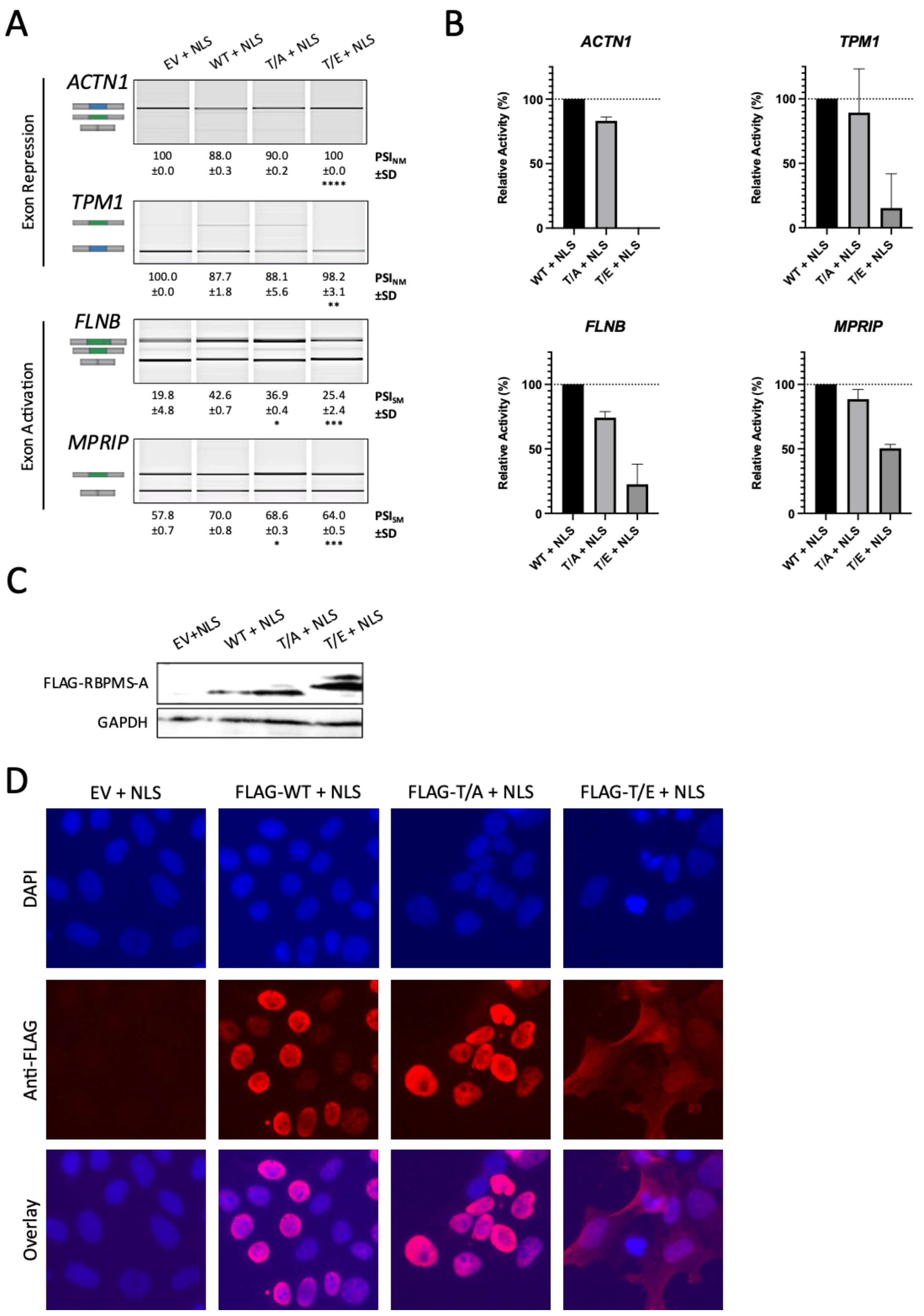

A
EV + NLS
WT + NLS
T/A + NLS
T/E + NLS
Exon Repression
ACTN1
100 ±0.0
88.0 ±0.3
90.0 ±0.2
100 ±0.0 ****
PSI NM ±SD
TPM1
100.0 ±0.0
87.7 ±1.8
88.1 ±5.6
98.2 ±3.1 **
Exon Activation
FLNB
19.8 ±4.8
42.6 ±0.7
36.9 ±0.4 *
25.4 ±2.4 ***
PSI SM ±SD
MPRIP
57.8 ±0.7
70.0 ±0.8
68.6 ±0.3 *
64.0 ±0.5 ***
B
ACTN1
TPM1
FLNB
MPRIP
Relative Activity (%)
0
50
100
WT + NLS
T/A + NLS
T/E + NLS
C
EV+NLS
WT + NLS
T/A + NLS
T/E + NLS
FLAG-RBPMS-A
GAPDH
D
EV + NLS
FLAG-WT + NLS
FLAG-T/A + NLS
FLAG-T/E + NLS
DAPI
Anti-FLAG
Overlay

Supplementary Figure S5. Exogenous NLS Fails to Rescue the Splicing Activity of RBPMS-A T/E.
(A) Splicing analysis of the exon repression function of wild-type (WT) RBPMS-A and effectors in endogenous *ACTN1* and *TPM1*, and its exon activation function in endogenous *FLNB* and *MPRIP* in HEK293T cells. RT-PCR followed by visualization on QIAxcel. Cartoons below the gene names represent the PCR products. The blue exon in the cartoon represents the NM (Non-smooth Muscle) exon, while the green exon represents the SM (Smooth Muscle) exon. PSI (Percent Spliced In) values are the mean ± SD (n = 3). A Student's t-Test was used to determine statistical significance between wild-type RBPMS-A and effector, indicated by *p < 0.05, **p < 0.01, ***p < 0.001, ****p < 0.0001.
(B) Bar graphs showing the percent relative splicing efficiency of each effector normalized to wild-type RBPMS-A (black bar), after background splicing was taken into consideration. Error bars indicate SD.
(C) Immunoblot of FLAG-RBPMS effectors used for splicing assays in (A). Whole-cell lysates were probed with antibodies to FLAG and GAPDH. GAPDH was used as a loading control.
(D) Immunofluorescence microscopy images of HEK293T cells overexpressing wild-type (WT) RBPMS-A and the T/A and T/E mutants all containing an exogenous nuclear localization signal (NLS). All images were taken with the same parameters using a 40X objective. Top row shows DAPI staining for DNA, middle row shows staining for FLAG-tagged effectors, and bottom row is an overlay of the DAPI and FLAG images.

Figure S6

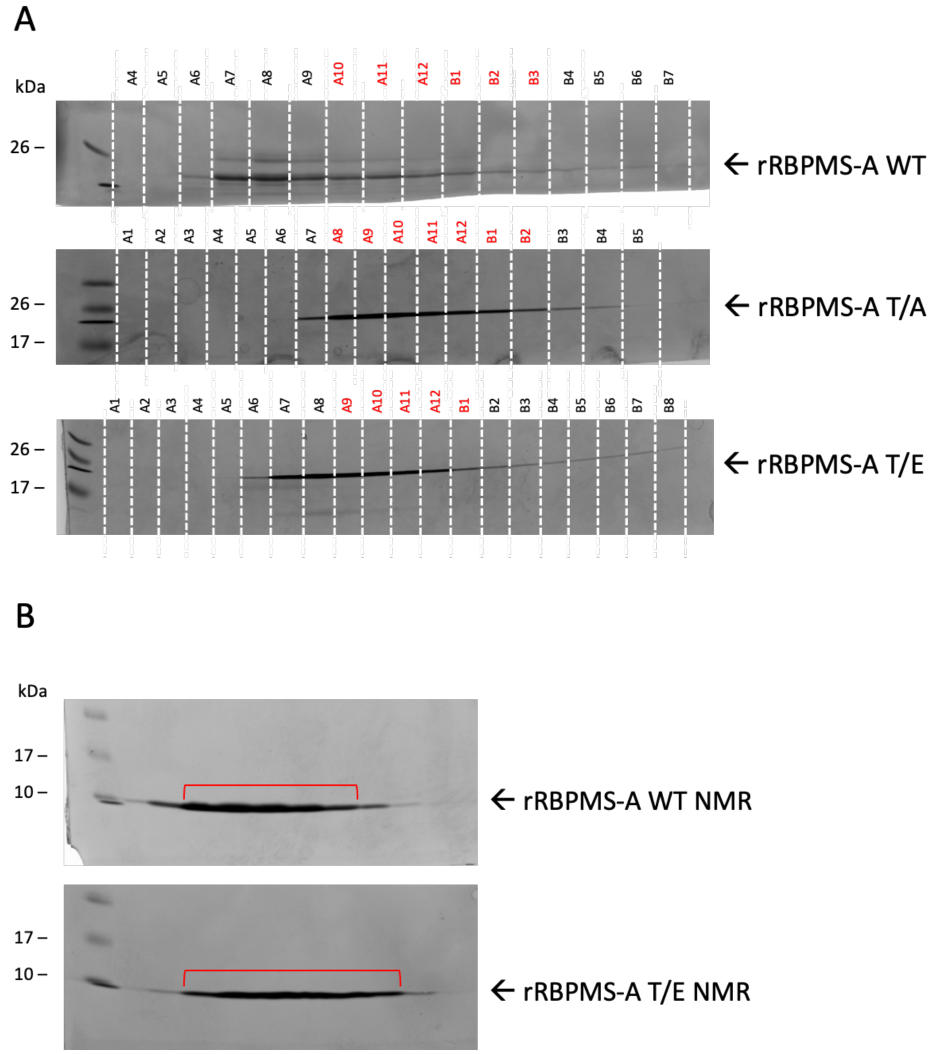

Supplementary Figure S6. Purification of Recombinant RBPMS-A.

(A) Final Mono Q™ anion exchange chromatography purification of recombinant RBPMS wild-type (WT), T/A, and T/E mutants following TEV protease treatment to remove His-tag. See methods for a detailed purification method. Fractions highlighted in red for each protein were pooled, quantified on a NanoDrop spectrometer, concentrations determined using predicted extinction coefficients, and used for follow on experiments.

(B) Final HiTrap™ Heparin chromatography purification of recombinant double-labeled ($^{13}C/^{15}N$) RBPMS-A aa1-122 WT and T/E mutant. See methods for detailed labeling and purification method. Fractions highlighted by the red bracket were pooled, quantified on a NanoDrop spectrometer, concentrations determined using predicted extinction coefficients, and used for follow-on NMR experiments.

Figure S7

A

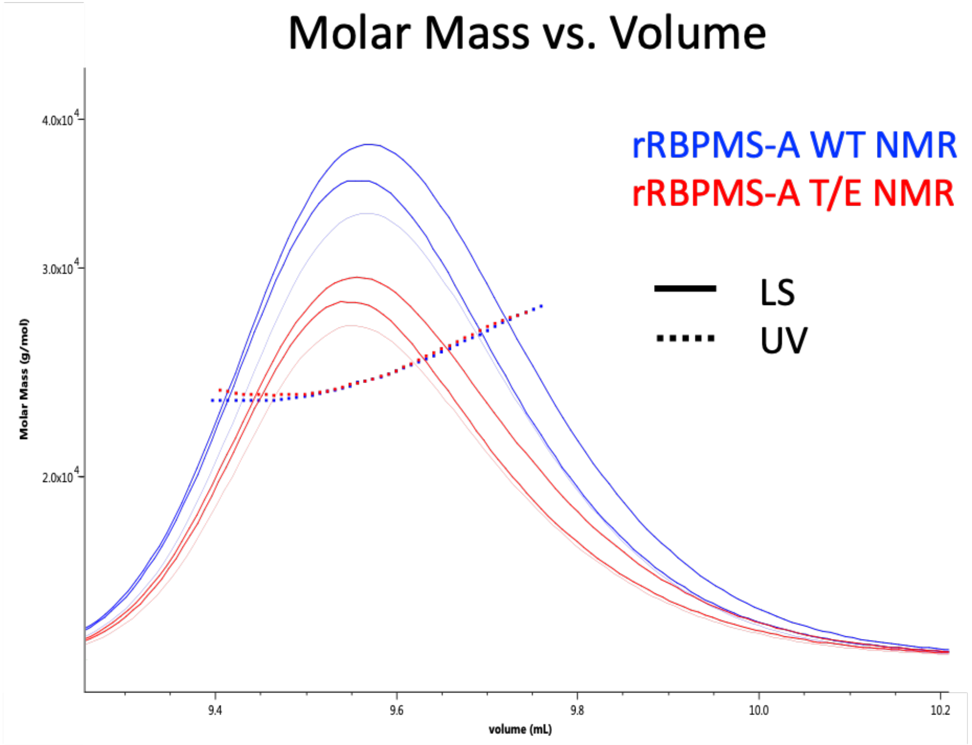

B

rRBPMS-A WT NMR

Peak Results	Peak 1
Masses	
Calculated Mass (µg)	317.87
Molar mass moments (g/mol)	
Mn	2.454×10^4 (±0.231%)
Mw	2.461×10^4 (±0.235%)
Polydispersity	
Mw/Mn	1.003 (±0.329%)
rms radius moments (nm)	
Rn	3.4 (±86.6%)
Rw	3.4 (±88.7%)
Rz	3.4 (±91.0%)

rRBPMS-A T/E NMR

Peak Results	Peak 1
Masses	
Calculated Mass (µg)	223.08
Molar mass moments (g/mol)	
Mn	2.455×10^4 (±0.115%)
Mw	2.461×10^4 (±0.113%)
Polydispersity	
Mw/Mn	1.002 (±0.161%)
rms radius moments (nm)	
Rn	2.7 (±59.5%)
Rw	2.7 (±61.4%)
Rz	2.6 (±63.6%)

Supplementary Figure S7. Truncated rRBPMS-A Proteins for NMR Remain Dimeric. Size exclusion chromatography multi-angle light scattering (SEC-MALS) of truncated NMR proteins. Conditions for both samples: 10 mM HEPES pH6.8 50 mM KCl. LS = light scattering; UV = ultraviolet absorbance (A) Chromatogram traces of each protein (B) MALS data output for each of the peaks in (A).

Figure S8

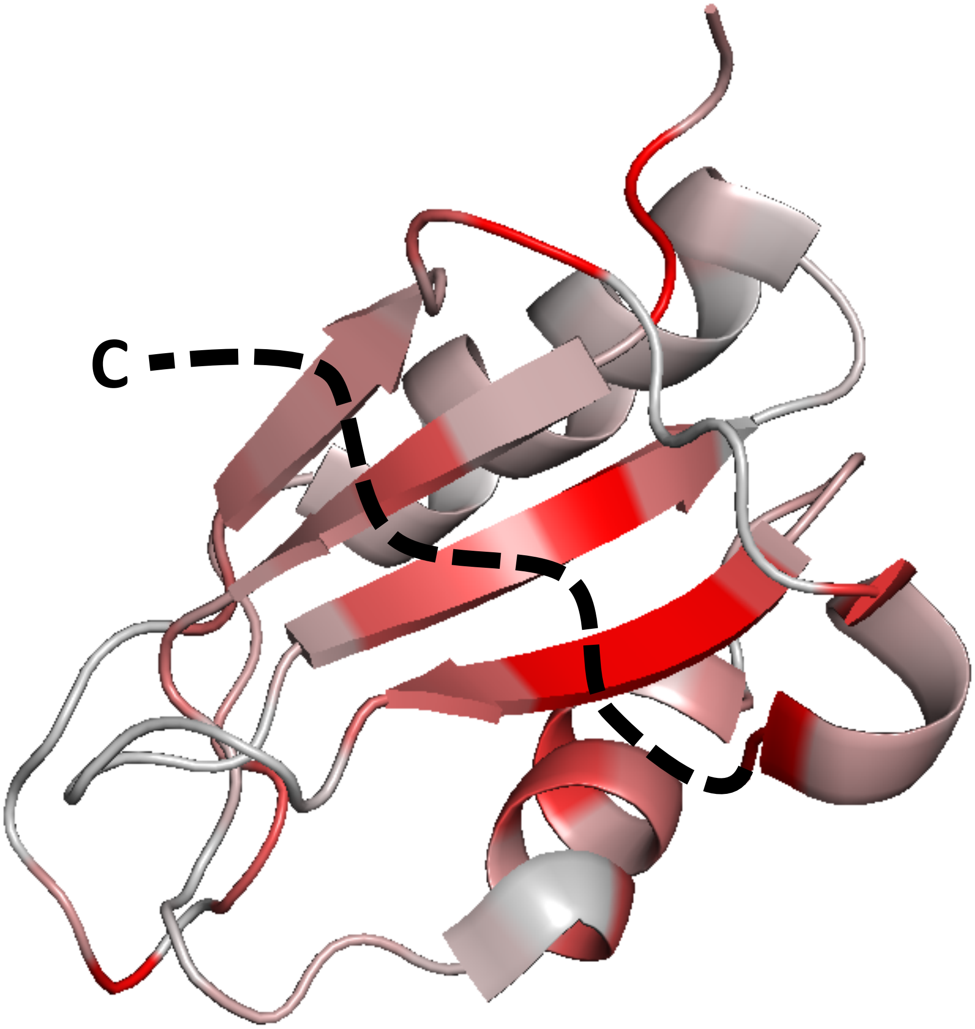

Supplementary Figure S8. Theoretical Model of C-Terminal Tail Looping for Phosphorylated (pT113/pT118) RBPMS-A

Proposed model of the negatively charged (pT113/pT118) C-terminal tail looping across the RBPMS-A RRM, acting as an RNA mimic and occluding RNA binding.
